# Herbal Medicine for the Treatment of Coronavirus Disease 2019 (COVID-19): A Systematic Review and Meta-Analysis of Randomized Controlled Trials

**DOI:** 10.3390/jcm9051583

**Published:** 2020-05-23

**Authors:** Lin Ang, Eunhye Song, Hye Won Lee, Myeong Soo Lee

**Affiliations:** 1 Clinical Medicine Division, Korea Institute of Oriental Medicine, Daejeon 34054, Korea; anglin2808@kiom.re.kr; 2 Korean Convergence Medicine, University of Science and Technology, Daejeon 34113, Korea; 3 Global Strategy Division, Korea Institute of Oriental Medicine, Daejeon 34054, Korea; esong@kiom.re.kr; 4 Department of Preventive Medicine, College of Korean Medicine, Daejeon University, Daejeon 34520, Korea; 5 Herbal Medicine Research Division, Korea Institute of Oriental Medicine, Daejeon 34054, Korea; hwlee@kiom.re.kr

**Keywords:** systematic review, complementary and alternative medicine, herbal medicine, coronavirus disease, COVID-19

## Abstract

Background: The coronavirus disease 2019 (COVID-19) pandemic has caused a worldwide outbreak of respiratory illness. This review aims to evaluate the effectiveness and adverse events of herbal medicines for the treatment of COVID-19. Methods: Twelve databases were searched through 12 May 2020. Randomized controlled trials (RCTs) and quasi-RCTs assessing the effects of herbal medicines for the treatment of COVID-19 were eligible. The study selection and data extraction were performed by two independent reviewers. The Cochrane risk of bias tool was used for the assessment of the risk of bias in all included RCTs. Mean differences (MDs), risk ratios (RRs) and odds ratios (ORs) with 95% confidence intervals (CIs) were calculated, and the effect sizes of the studies were pooled. Results: Seven RCTs with a total of 855 patients were included. All included trials compared the combined therapy of herbal medicine with Western medicine to Western medicine alone. The combined therapy significantly improved the total effective rate (RR 1.23, 95% CI 1.13 to 1.34, *p* < 0.001), cough symptom disappearance rate (RR 1.45, 95% CI 1.12 to 1.89, *p* = 0.005), and sputum production symptom disappearance rate (RR 1.73, 95% CI 1.19 to 2.50, *p* = 0.004). Beneficial effects of the combined therapy were also seen in TCM syndrome score of cough (MD −1.18, 95% CI −1.34 to −1.03, *p* < 0.001), fever (MD −0.62, 95% CI −0.79 to −0.45, *p* < 0.001), dry and sore throat (MD −0.83, 95% CI −1.45 to −0.20, *p* = 0.009), and fatigue (MD −0.60, 95% CI −1.04 to −0.17, *p* = 0.007). The overall risk of bias of the included studies was unclear. No serious adverse events were reported. Conclusion: Significant effects of the combined therapy of herbal medicine with Western medicine were found, and revealed the potential role of herbal medicine in treating COVID-19. More high-quality RCTs are needed to further validate the effectiveness and adverse events of herbal medicine in the treatment of COVID-19.

## 1. Introduction

Beginning in December 2019, a novel coronavirus disease, COVID-19, also referred to as SARS-CoV-2, has caused an international outbreak of acute respiratory illness. The rapid spread of COVID-19 was characterized as a pandemic by the World Health Organization on 11 March 2020 [1]. This pandemic has affected at least 177 countries, with approximately 154,000 fatalities [2]. Currently, there are no specific therapeutic agents for this disease, due to its broad clinical spectrum.

In the past, herbal medicine has played an important role in controlling infectious diseases. Clinical evidence from a range of studies of herbal medicine in the treatment of SARS coronavirus (SARS-CoV) has shown significant results, and supported the idea that herbal medicine has a beneficial effect in the treatment and prevention of epidemic diseases [3]. A Cochrane systematic review reported that herbal medicine combined with Western medicine may improve symptoms and quality of life in SARS-CoV patients [4]. A recently conducted meta-analysis also concluded that herbal medicine could reduce the infection rate of H1N1 influenza [5].

Inspired by previous experience, herbal medicine is considered one of the alternative approaches in the treatment of COVID-19. In China, the National Health Commission has declared the use of herbal medicine combined with Western medicine as a treatment for COVID-19, and has issued many guidelines on herbal medicine-related therapy [6]. To date, there is much clinical evidence that reports favorable effects of the usage of herbal medicine in the treatment of COVID-19 [7]. Several systematic reviews that included evidence from case reports, case series, and observational studies have also been conducted, to study the effectiveness of herbal medicine in the treatment of COVID-19 [8,9,10]. However, in the hierarchy of systematic reviews, reviews of randomized control trials (RCTs) offer the highest level of evidence. 

Thus, in this review, we aimed to evaluate the effectiveness and adverse events of herbal medicines in the treatment of COVID-19, based only on currently available RCTs.

## 2. Methods

### 2.1. Study Registration

This review was conducted in accordance with the Preferred Reporting Items for Systematic Reviews and Meta-Analyses (PRISMA) guidelines [11]. The protocol of this review was previously registered with the Research Registry (unique identifying number: researchregistry872).

### 2.2. Search Strategy

A systematic literature search was then performed by two authors, by searching the following electronic bibliographic databases:

- English databases: PubMed, Embase, Allied and Complementary Medicine Database (AMED) and Cochrane Register of Controlled Trials (CENTRAL).
- Chinese databases: Chinese National Knowledge Infrastructure Database (CNKI), Chinese Science and Technique Journals Database (VIP), Chinese Biomedical Literature Database (CBM) and the Wanfang Database.
- Korean databases: Korean Association of Medical Journal database (KoreaMed), Korean Medical database (KMBase), Research Information Service System (RISS), and OASIS database.

All databases were searched from the available date of inception through 12 May 2020. The search strategy included the following terms: (“coronavirus disease 2019” OR “COVID-2019” OR “2019 novel coronavirus” OR “2019-nCoV” OR “Novel Coronavirus Pneumonia” OR “NCP” OR “Severe acute respiratory syndrome coronavirus 2” OR “SARS CoV-2” OR “new coronavirus” OR “novel coronavirus”) AND (“herbal medicine” OR “traditional medicine” OR “oriental medicine” OR “Chinese medicine” OR “Korean medicine” OR “herbal formula” OR herb). Any indexed terms equivalent to “COVID-2019” and “herbal medicine” were also searched to extend the search coverage.

We also searched the National Institute of Health and Clinical Trials Database (http://www.clinicaltrials.gov/), WHO’s International Clinical Trials Registry Platform (https://www.who.int/ictrp/en/), Chinese Clinical Trial Registry (http://www.chictr.org.cn/) and for any ongoing clinical trials. There were no restrictions concerning language or publication type. Two authors independently screened the titles and abstracts for eligibility. All searches were reconducted before the completion of this review, to retrieve any further includable studies.

### 2.3. Eligibility Criteria

#### 2.3.1. Types of Studies

Studies were eligible if they were randomized controlled trials (RCTs) or quasi-RCTs that included herbal medicine as a treatment for COVID-19. Studies such as case-control studies, cohort studies, case reports, and animal and experimental studies were excluded. There were no restrictions regarding language or publication status.

#### 2.3.2. Participants

We included participants who (1) were diagnosed with COVID-19, regardless of their age, sex and ethnicity; (2) presented positive RT-PCR nucleic acid test results; (3) did not have immediate life-threatening comorbidities; (4) did not use herbal medicine for other chronic disorders, or for any other purpose; and (5) were pregnant or breastfeeding.

#### 2.3.3. Intervention Groups

Any forms of oral administration of herbal medicine treatment, including herbal decoctions or patent medicine, were included. Combined interventions using herbal medicine and Western medicine were also eligible. Non-herbal medicine interventions, herbal injections, or combined interventions between two or more different types of herbal medicine were excluded. There were no limitations regarding the composition of herbal medicine, the intake dosage or frequency, or the treatment duration.

#### 2.3.4. Comparison Groups

Comparison groups that received no treatment or only Western medications for COVID-19 treatment were included. Placebo groups were also eligible for comparison groups. Comparator groups that involve different types of herbal medicine or herbal medicine of the same type with different dosages were excluded.

#### 2.3.5. Outcome Measures

The primary outcomes comprised the total effective rate, the symptom score, and symptom disappearance rate. Both effective rate and the symptom score were evaluated, according to the “Guideline of clinical new drug research in Chinese herbal medicine” [12]. The effective rate was defined as the number of patients whose total symptom score reduce greater than or equal to 30 percent after treatment. The symptom score, also referred as TCM syndrome score in this review, was defined as the score of common TCM clinical symptoms, which can be scored as 0 points (no symptom), 1 point (mild), 2 points (moderate), or 3 points (severe). Additionally, the core outcome set of clinical trials (COS-COVID) was also assessed as primary outcomes in this review [13].

There were no restrictions on secondary outcomes. Any relevant clinical outcomes, such as blood test results (complete blood count), duration of symptoms, changes in chest CT scans, quality of life (using a validated instrument), and adverse events, were eligible for inclusion. 

### 2.4. Study Selection and Data Extraction

Two review authors (LA and ES) independently searched the databases and assessed the eligibility of the studies after removing duplicates. The full text versions of the potentially eligible studies were then obtained and screened, based on the inclusion criteria. Any discrepancies in the suitability of a study for inclusion in this review were discussed with a third review author (MSL), until a consensus was reached.

Subsequently, two independent review authors (LA and ES) extracted the data using a standard data extraction form. The following information was extracted: authors’ first name, publication year, country, intervention model, sample size, patient age and sex, duration and stage of the disease, details of the interventions and controls (regimens), outcome measures, study results, and adverse events. All disagreements between the two authors’ judgments were resolved with the third review author (MSL) through discussion. The authors of the included studies were contacted for unreported data or missing data.

### 2.5. Assessment of Risk of Bias

Two review authors (LA and ES) individually assessed the risk of bias of the included studies using the Cochrane Collaboration’s Risk of Bias Assessment tool) [14]. The following six items were assessed: the generation of a random sequence, concealment of allocation, blinding of participants and investigators, incomplete outcome data, selective outcome reporting, and other possible biases. The risk of bias of each item was categorized into low, unclear, or high risk. The overall risk of bias of the included studies was also assessed. Any disagreements over the risk of bias in a particular study were resolved through the involvement of a third party.

### 2.6. Data Analysis

All data were analyzed using Review Manager (RevMan) version 5.3 software (The Nordic Cochrane Centre, The Cochrane Collaboration, Copenhagen, Denmark). The risk ratios (RRs) or odds ratios (ORs) with 95% confidence intervals (CIs) were calculated for dichotomous data (e.g., symptom disappearance rate and effective rate), while the mean differences (MDs) with 95% CIs were calculated for continuous data (e.g., symptom scores). As the variability between the included studies was taken into consideration, the random-effects model was used to pool the data. The heterogeneity levels of the eligible RCTs were assessed using *I*^2^ statistics. As there were only a few studies included in this review, subgroup analysis was not performed.

## 3. Results

### 3.1. Literature Search

**Figure 1:**
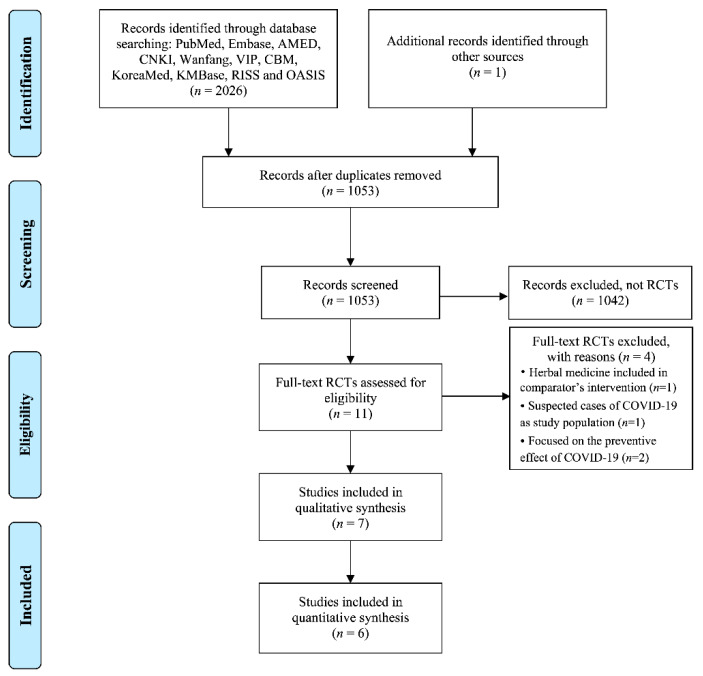
Flowchart of the literature search and study selection. COVID-19: coronavirus disease 2019; RCT: randomized controlled trial.

The database search identified 2027 studies, as shown in Figure 1. We screened the titles and abstracts of 1053 studies after removing duplicates, and another 1042 articles were excluded, because they were not RCTs. Only 11 RCTs were found, and the full articles of studies were then retrieved. Based on our predefined inclusion and exclusion criteria, four RCTs were further excluded, where one RCT [15] included herbal medicine as a comparator intervention, one RCT studied on suspected cases of COVID-19 [16], and two RCTs focused on the preventive effect [17,18]. A total of seven studies were hence included in this review [19,20,21,22,23,24,25]. We did not find any further includable studies after reconducting all searches, before the completion of this review.

### 3.2. Study Characteristics

The characteristics of the included studies are presented in Table 1. All RCTs were conducted in mainland China. Four trials included patients in the mild or moderate stage of the disease, and the other three trials included only patients in the mild, moderate, and severe stage of the disease in each trial respectively. The sample size was 855 in total (ranging from 42 to 295), with 472 (55%) male participants and 383 (45%) female participants. Mean age was 50.5 years (ranging from 42.0 to 65.0 years).

All trials were parallel-design trials and applied combined therapy of herbal medicine with Western medicine for COVID-19 treatment. Herbal medicine intervention in the included trials were given orally which included Chinese patent medicine (Lianhua Qingke granules, Shufeng Jiedu capsule, Jinhua Qinggan granules, Toujie Quwen granules) and herbal decoction (prescribed according to pattern identification as shown in Supplementary Table S1). The comparators of the trials only included Western medicines. Types of Western medicines included were as follows: Lopinavir/ritonavir, Arbidol Hydrochloride tablets, Chloroquine Phosphate tablets, Ambroxol Hydrochloride tablets, Moxifloxacin tablets, Interferon-alfa injections, and Ribavirin injections. No study compared monotherapy of herbal medicine to no treatment, or monotherapy of Western medicine.

### 3.3. Risk of Bias Assessment

**Figure 2:**
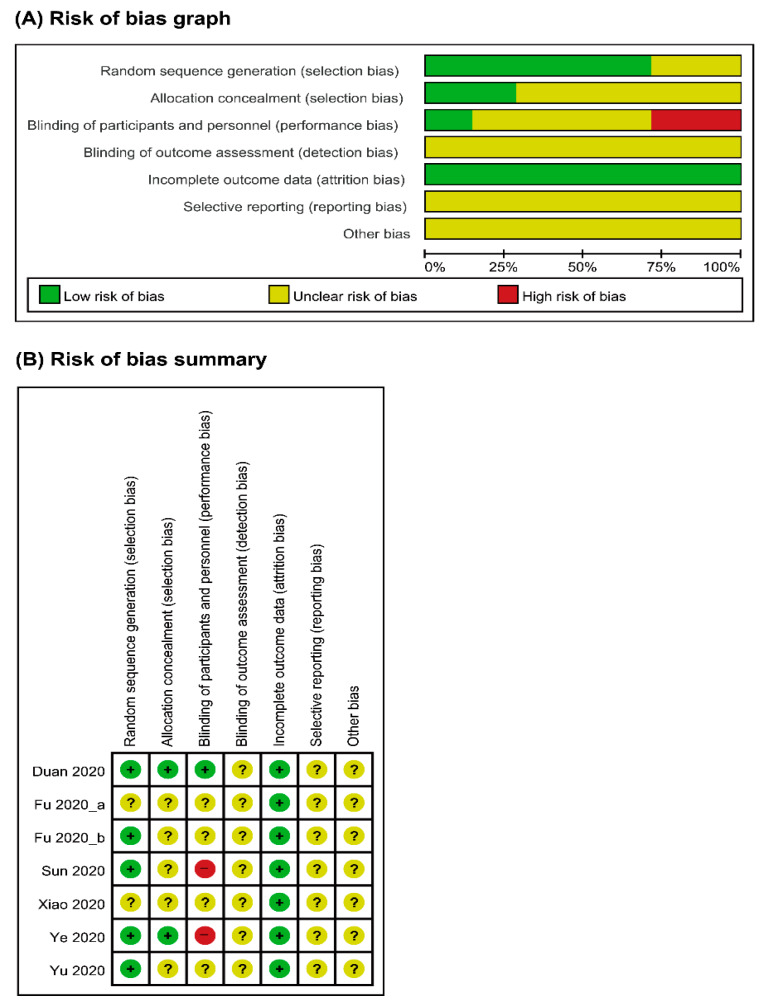
Risks of bias. (**A**) Risks of bias of the included studies. The authors reviewed each item’s risk of bias for each included study. (**B**) Risks of bias of individual studies. +: low risk of bias; −: high risk of bias; ?: unclear risk of bias.

Overall, the risk of bias of the included studies was considered unclear. Five studies [19,21,23,24,25] reported adequate random sequence generation, but the other two studies [20,22] did not describe the method of randomization. Except for two studies [21,25], none of the studies provided information on allocation concealment. The performance bias was unclear for four studies [20,22,23,24], as this information was not provided; one study [21] was a single-blinded trial, and the remaining two studies [19,25] were open-label trials. The risk of bias for incomplete outcome data was evaluated as low for all studies [19,20,21,22,23,24,25], on the grounds of having no dropouts in four studies [19,20,22,23] and of performing intention-to-treat analysis in three studies [21,24,25]. However, the risk of selective reporting was unclear for all studies [19,20,21,22,23,24,25], as there was not enough information to reach judgment, and there was no study protocol available for most studies. One study [25] with study protocol was also assessed as unclear risk in reporting bias due to missing outcome data. The risk of other biases was considered unclear for all studies [19,20,21,22,23,24,25], due to the small sample size, short study duration, and lack of information on sources of funding. The summaries of the risk of bias assessment are illustrated in Figure 2.

### 3.4. Effect of Intervention on Primary Outcomes

#### 3.4.1. Primary Outcomes

(1) Total Effective Rate

**Figure 3:**
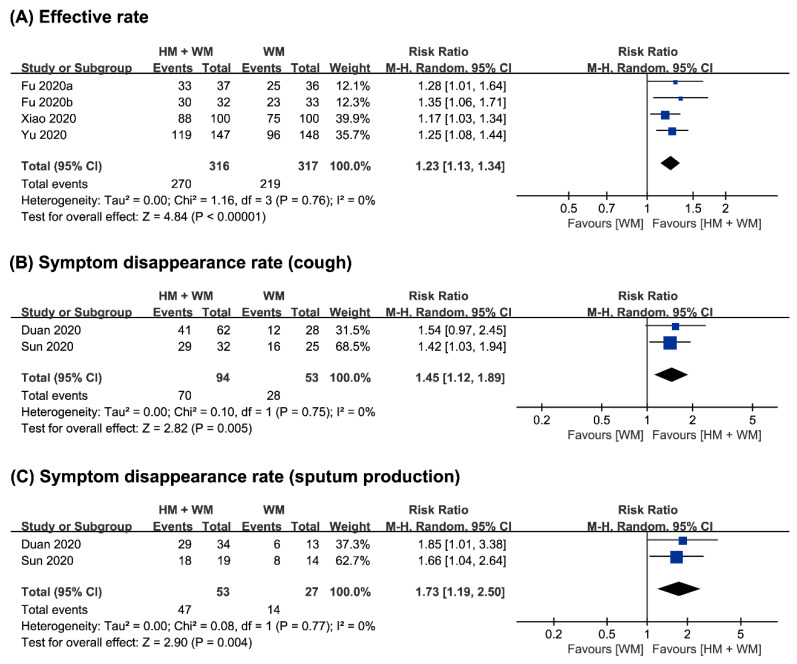
Comparison of herbal medicine and Western medicine (HM + WM) vs. Western medicine (WM) on (**A**) the total effective rate; (**B**) symptom disappearance rate of cough; and (**C**) sputum production.

Four studies [20,22,23,24] assessed the total effective rate of the treatment of COVID-19. The combined therapy of herbal medicine with Western medicine showed a significantly greater effect with regard to the effective rate (*n* = 633, RR 1.23, 95% CI 1.13 to 1.34, *p* < 0.001, Figure 3A). 

(2) Symptom Disappearance Rate

Two studies [19,21] evaluated the symptom disappearance rate between groups. One study [19] showed that the disappearance rate of cough and sputum production showed significant improvements after the intervention. Another study [21] also showed significant improvements in the disappearance rate of fever, fatigue, cough, sputum production, and diarrhea after the intervention. Meta-analysis showed a significant effect of combined therapy on the disappearance rate of cough after the intervention (*n* = 147, RR 1.45, 95% CI 1.12 to 1.89, *p* = 0.005, Figure 3B). The combined therapy also showed a positive effect on the disappearance rate of sputum production (*n* = 80, RR 1.73, 95% CI 1.19 to 2.50, *p* = 0.004, Figure 3C).

(3) Symptom Score

**Figure 4:**
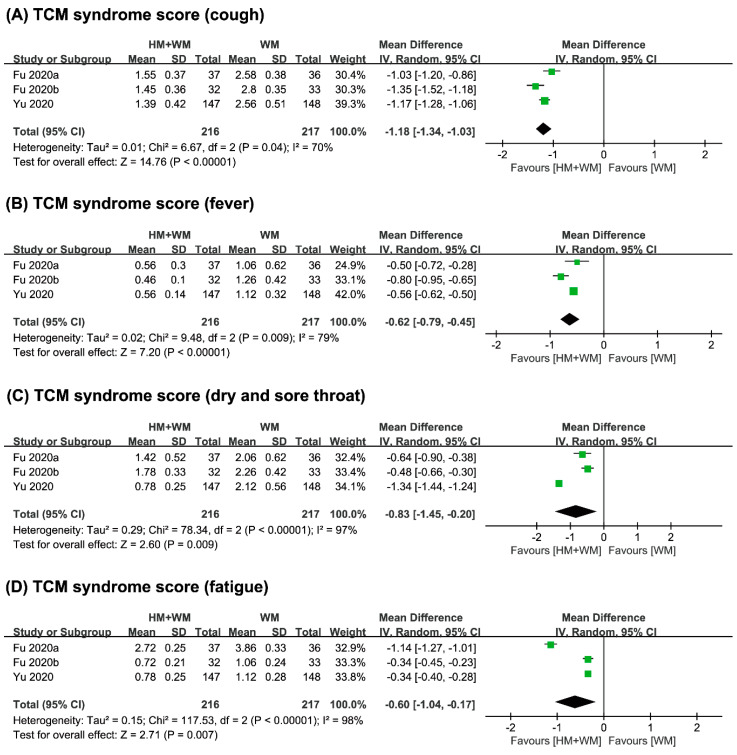
Comparison of herbal medicine and Western medicine (HM + WM) vs. Western medicine (WM) on TCM syndrome score of (**A**) cough; (**B**) fever; (**C**) dry and sore throat; (**D**) fatigue.

The symptom score outcomes in the studies included in this review were measured using the TCM syndrome score. Four studies [21,22,23,24] assessed the TCM syndrome score. The combined therapy of herbal medicine with Western medicine showed favorable results with regard to the total syndrome score in a study [21] (MD 1.39 (−0.21, 2.99), *p* < 0.05). The other three studies [22,23,24] also showed favorable results towards the combined therapy, but they only reported the syndrome score for different clinical symptoms (all symptoms, *p* < 0.05; Table 1). Meta-analysis showed beneficial effects of combined therapy on TCM syndrome score of cough (*n* = 433, MD −1.18, 95% CI −1.34 to −1.03, *p* < 0.001, Figure 4A), fever (*n* = 433, MD −0.62, 95% CI −0.79 to −0.45, *p* < 0.001, Figure 4B), dry and sore throat (*n* = 433, MD −0.83, 95% CI −1.45 to −0.20, *p* = 0.009, Figure 4C), and fatigue (*n* = 433, MD −0.60, 95% CI −1.04 to −0.17, *p* = 0.007, Figure 4D). 

#### 3.4.2. Secondary Outcomes 

(1) Changes in Blood Test Results

**Figure 5:**
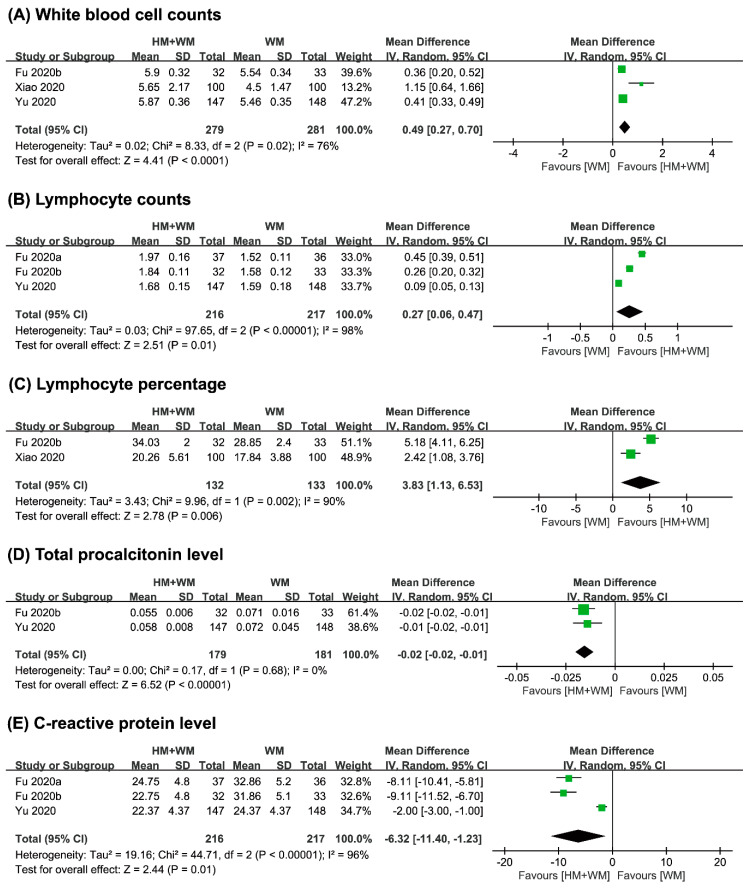
Comparison of herbal medicine and Western medicine (HM + WM) vs. Western medicine (WM) on (**A**) white blood cell counts (10^9^ cells/L); (**B**) lymphocyte counts (10^9^ cells/L); (**C**) lymphocyte counts percentage; (**D**) total procalcitonin level (ng/L); (**E**) C-reactive protein (mg/L).

Five studies [20,22,23,24,25] performed a routine blood test on the patients after the intervention, to observe the changes in the complete blood count. In comparison to Western medicine, the combined therapy of herbal medicine with Western medicine in three studies [20,22,23] showed a significantly greater effect in increasing white blood cell counts (*n* = 540, MD 0.49, 95% CI 0.27 to 0.70, *p* < 0.001, Figure 5A). Two studies [20,23] also showed beneficial effect on combined therapy for lymphocyte percentage (*n* = 265, MD 3.83, 95% CI 1.13 to 6.53, *p* = 0.006, Figure 5C). A high heterogeneity (*I*^2^ > 76%) observed in the pooled results of both outcomes (Figure 5A,C).

On the other hand, three studies [22,23,24] reported on lymphocyte counts and C-reactive protein level. Meta-analysis showed favorable effects of combined therapy for both lymphocyte counts (*n* = 433, MD 0.27, 95% CI 0.06 to 0.47, *p* < 0.001, Figure 5B) and C-reactive protein level (*n* = 433, MD−6.32, 95% CI −11.40 to −1.23, *p* < 0.001, Figure 5E). A high heterogeneity (*I*^2^ > 96%) was observed in the pooled results of these outcomes (Figure 5B, E). Outcomes such as procalcitonin level were also reported by two studies [23,24], where the pooled effects also showed positive effects of the combined therapy after intervention (*n* = 360, MD −0.02, 95% CI −0.05 to −0.01, *p* < 0.001, Figure 5D). 

Only one study [23] reported on the neutrophil percentage and D-Dimer level after the intervention, and showed significant results towards the combined therapy of herbal medicine with Western medicine (*n* = 65, neutrophil percentage, MD −4.58, 95% CI −5.81 to −3.35, *p* < 0.05; D-Dimer level, MD −42.50 95% CI −84.55 to −0.45, *p* < 0.05).

(2) Duration of Symptoms and Quality of Life (QOL) Assessment

One study [21] evaluated the duration of symptoms after the intervention, according to symptom disappearance time. In comparison to Western medicine, the combined therapy of herbal medicine with Western medicine only showed a beneficial effect on the symptom disappearance time for fever (*n* = 123, RR 1.51, 95% CI 1.07 to 2.14, *p* < 0.05), while the symptom disappearance time for other symptoms, such as cough, fatigue, vertigo, nasal congestion, and rhinorrhea, was not significant.

None of the included studies assessed the quality of life of the patients after the intervention. Only one study [21] assessed the anxiety of patients based on the Hamilton Anxiety Scale. The results showed that the combined therapy of herbal medicine with Western medicine had a significant effect on relieving anxiety in patients (*n* = 123, MD 0.34, 95% CI −1.14 to 1.82, *p* < 0.01).

(3) Changes in Chest CT Scans and Oxygenation Index

Three studies [19,23,24] examined the improvement of abnormalities in chest CT, where one study [18] showed positive effect towards the combined therapy of herbal medicine with Western medicine (*n* = 57, RR 1.35, 95% CI 1.05 to 1.73, *p* < 0.05), and the other two studies [23,24] reported no significance. One study [19] that measured the oxygenation index (*n* = 57, MD 73.73, 95% CI 52.75 to 94.71, *p* < 0.05) and another study [20] that assessed the absorption of lesions based on chest CT (*n* = 200, RR 1.21, 95% CI 1.05 to 1.40, *p* < 0.05) after the intervention also obtained favorable results for both outcomes. 

(4) Hospital Discharge Rate and Composite Events

One study [22] assessed the hospital discharge rate and reported that the number of patients discharged from the combined therapy of herbal medicine with Western medicine group was significantly higher than the Western medicine group (*n* = 73, RR 1.42, CI 95% 0.76 to 2.62, *p* < 0.05). In terms of composite events, there were no significant differences reported by the only study [25] that evaluated the changes in the disease severity, the overall survival through last day of treatment, the proportion of patients without improvement, and the prevalence of antibiotic use during treatment for both the intervention and control groups. 

(5) Adverse Events (AEs)

**Figure 6:**
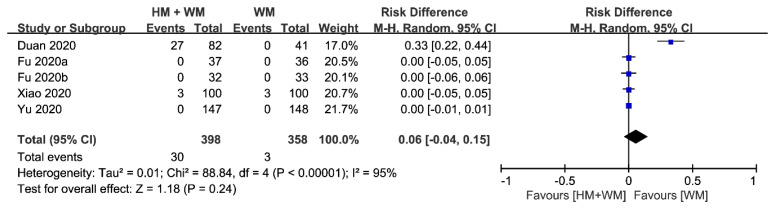
Comparison of herbal medicine and Western medicine (HM + WM) vs. Western medicine (WM) on occurrence of adverse events.

Adverse events of the combined therapy of herbal medicine with Western medicine were compared to those of Western medicine in five studies [20,21,22,23,24]. Two studies [20,21] reported the occurrence of minor AEs, three studies [22,23,24] reported no AEs at all, and two studies [19,25] did not assess AEs. Minor AEs were equally distributed in one study [20], and more AEs were observed in the combined therapy group in the other study [21]. Among these two studies, one study [20] did not provide information on withdrawn cases and the other study [21] stated that there were eight cases of withdrawals due to AEs. The pooled effects of the five studies [20,21,22,23,24] were not statistically significant (*n* = 756, Risk difference, 0.06, 95% CI −0.04 to 0.15, *p* = 0.24, Figure 6) with high heterogeneity (*I*^2^ = 95%).

## 4. Discussion

### 4.1. Summary of Evidence

The systematic search revealed only seven RCTs investigating the effectiveness of herbal medicine for COVID-19 treatment. In comparison to Western medicine, the meta-analysis showed significant effects of the combined therapy of herbal medicine with Western medicine after intervention for the total effective rate, the disappearance rate (cough and sputum production), TCM syndrome score (cough, fever, dry and sore throat, and fatigue), and complete blood count (white blood cell and lymphocyte counts, lymphocyte percentage, and level of procalcitonin and C-reactive protein).

The risk of bias of the included RCTs was unclear in general, resulting in a limitation in drawing a reliable conclusion on the effectiveness of herbal medicine in the treatment of COVID-19. On the other hand, no serious AEs were reported. Minor AEs were reported less often in the Western medicine group than in the intervention group of the combined therapy of herbal medicine with Western medicine. Nevertheless, the AEs stated in the included studies were not sufficient to provide a report on the adverse events of herbal medicine used to treat COVID-19.

To conduct a more comprehensive systematic review in the future, we searched for all ongoing RCTs for possible inclusion in our review, on the basis of our predefined criteria. We found 32 ongoing RCTs that were eligible for our future review. We summarize the eligible studies on the herbal treatment of COVID-19, which are still under clinical investigation, in Table 2.

### 4.2. Advances in the Prior Systematic Review

In comparison to prior systematic reviews that included case reports, case-control, and cohort studies [8]; reviewed only case reports and case series [10]; and focused only on one type of herbal medicine [9], this review focused only on RCTs investigating herbal medicine treatments in COVID-19 patients. Thus, we performed the first meta-analysis of RCTs on herbal medicine interventions, although the analysis could only be conducted on limited studies.

### 4.3. Limitations of This Review

First, the small number of studies included in this review was our largest limitation. Due to the small number of included studies, the studies that could be included in the meta-analysis are highly restricted. The significance of the results may change with the inclusion of additional studies. Second, the risk of bias assessment of the included studies was unclear. Many studies did not report on the generation of sequences, concealment of allocation, or blinding of participants and study investigators, or provide alternative methods used to reduce potential performance bias. As we included all the studies in our analysis, the results of our analysis might have a certain degree of bias. Third, clinical studies on COVID-19 are still in their early stage overall, as the outbreak is considered recent. Hence, there are very few publications of RCTs related to herbal medicine treatments, and most publications are from mainland China. This review may be considered less informative, and the results of our review may be difficult to generalize.

### 4.4. Implications for Clinical Practice

The summarized evidence in this review showed the potential of herbal medicine for treating COVID-19. The combined therapy of herbal medicine with Western medicine has shown significant results in increasing the effective rate and improving the symptoms disappearance rate, TCM syndrome score and complete blood count, compared to the effects of Western medicine monotherapy. The integration of both herbal and Western medicine could be an alternative for reducing the duration of treatment and increasing the speed of recovery. However, we cannot provide a recommendation, as the evidence of our review was obtained from limited studies.

### 4.5. Implications for Further Research

Further RCTs on herbal medicine for the treatment of COVID-19 are still urgently needed. This review provides existing evidence that might help to shape the design of future trials. Although double‒blinded trials may be difficult due to the nature of the disease, study investigators should consider alternative strategies to minimize the risk of performance bias. The trials could have also at least blinded the individuals who assessed the trial outcomes. After incorporating these methodologic precautions, study investigators should acknowledge the potential biases arising from the lack of blinding, and address them appropriately in the limitations of their study. In addition, study investigators may also refer to developed core outcome sets, such as COS-COVID [13], for their outcome measures, to avoid the waste of research resources. Regardless, both study investigators and authors should ensure a strict methodology and proper reporting, to reduce potential biases in trials evaluating the effectiveness of herbal medicine for the treatment of COVID-19.

## 5. Conclusions

Our results showed significant effects of the combined therapy of herbal medicine with Western medicine on the effective rate and improvement of symptoms. This reveals the potential role of herbal medicine in treating COVID-19. More high-quality RCTs are needed to further corroborate the effectiveness and adverse events of herbal medicine in the treatment of COVID-19.

## Figures and Tables

**Table 1:** Summary of included studies.

Author (Year) [Ref]	Sample Size (M/F) Disease Stages * Age (Years) Disease Course (Days)	Intervention (Regimen)	Control (Regimen)	Study Outcomes	Results
Sun (2020) [19]	57 (28/29) Mild, moderate A: 45.4; B: 42.0 A: 11.7; B: 13.0	(A) HM (Lianhua Qingke granules, 1 packet for 3 times daily for 14 days, *n* = 32), plus B	(B) WM (Lopinavir/ Ritonavir + Alpha interferon injection for 2 times daily, *n* = 25)	(1) Symptom disappearance rate (2) Improvement of abnormalities in chest CT (3) Oxygenation index	(1) Cough, RR 1.42 (1.03, 1.94), *p* < 0.05; sputum production, RR 1.66 (1.04, 2.64), *p* < 0.05; fever, RR 1.00 (0.68, 1.46), NS; fatigue, RR 1.25 (0.90, 1.75), NS; dry throat, RR 1.31 [0.62, 2.80], NS; sore throat, RR 1.00 (0.62, 1.60), NS. (2) RR 1.35 (1.05, 1.73), *p* < 0.05. (3) MD 73.73 (52.75, 94.71), *p* < 0.05.
Xiao (2020) [20]	200 (130/70) Mild, moderate A:60.9; B: 60.2 A: 5.5; B:6.4	(A) HM (Shufeng Jiedu capsule, 4 capsules for 3 times daily for 2 weeks, *n* = 100), plus B	(B) WM (Arbidol Hydrochloride tablets, 200mg for 3 times daily, *n* = 100)	(1) Effective rate (2) Symptom disappearance time (day) (3) Changes in WBC cell counts and LYM% (4) Absorption of lesions >50% based on chest CT scans	(1) RR 1.17 (1.03, 1.34) *p* < 0.05. (2) Fever, MD −0.83 (−1.22, −0.44), *p* < 0.05; cough, MD 0.28 (−0.40, 0.96), NS; fatigue, MD −0.33 (−0.78, 0.12), NS; vertigo, MD 0.18 (−0.31, 0.67), NS; nasal congestion, MD −0.17 (−0.61, 0.27), NS; rhinorrhea, MD 0.08 (−0.33, 0.49), NS. (3) WBC cell counts, MD 1.15 (0.64, 1.66), *p* < 0.05; LYM%, MD 2.42 (1.08, 3.76), *p* < 0.05. (4) RR 1.21 (1.05, 1.40), *p* < 0.05.
Duan (2020) [21]	123 (62/61) Mild A: 52.0; B: 50.3 A: 2.7; B: 2.5	(A) HM (Jinhua Qinggan granules, 2 packets for 3 times daily for 5 days, *n* = 82), plus B	(B) WM (Lopinavir/Ritonavir, 200 mg + Chloroquine Phosphate tablets, 500 mg + Alpha interferon and ribavirin injection for 2 times daily + Arbidol Hydrochloride tablets, 500 mg for 3 times daily, *n* = 41)	(1) Symptoms disappearance rate (2) Total TCM syndrome score (3) Hamilton Anxiety Scale	(1) Fever, RR 1.51 (1.07, 2.14), *p* < 0.05; chill, RR 0.99 (0.74, 1.34), NS; myalgia, RR 1.17 (0.73, 1.87), NS; heavy head and limbs, RR 0.84 (0.60, 1.19), NS; fatigue, RR 1.44 (0.98, 2.11), *p* < 0.05; cough, RR 1.54 (0.97, 2.45), *p* < 0.05; sputum production, RR 1.85 (1.01, 3.38), *p* < 0.05; sore throat, RR 1.30 (0.58, 2.87), NS; itchy throat, RR 1.14 (0.60, 2.17), NS; dry throat, RR 0.87 (0.54, 1.42), NS; nasal congestion or rhinorrhea, RR 1.31 (0.57, 3.05), NS; nausea or vomiting, RR 1.17 (0.69, 1.99), NS; diarrhea, RR 0.06 (0.00, 1.03), *p* < 0.05. (2) MD 1.39 (−0.21, 2.99), *p* < 0.05. (3) MD 0.34 (−1.14, 1.82), *p* < 0.01.
Fu (2020a) [22]	73 (38/35) Moderate A: 45.3; B: 44.7 A: 7.6; B: 8.5	(A) HM (Toujie Quwen granules, 1 packet per time for 2 times daily for 15 days, *n* = 37), plus B	(B) WM (Arbidol Hydrochloride tablets, 200 mg + Ambroxol Hydrochloride tablets, 30 mg for 3 times daily, *n* = 36)	(1) Effective rate (2) TCM syndrome score (3) Changes in WBC cell counts, LYM cell counts, LYM% and CRP level (4) Hospital discharge rate	(1) RR 1.28 (1.01, 1.64), *p* < 0.05. (2) Fever, MD −0.50 (−0.72, −0.28), *p* < 0.05; Cough, MD −1.03 (−1.20, −0.86), *p* < 0.05; Dry throat and sore throat, MD −0.64 (−0.90, −0.38), *p* < 0.05; Chest tightness and shortness of breath, MD −0.18 (−0.37, 0.01), *p* < 0.05; Fatigue, MD −1.14 (−1.27, −1.01), *p* < 0.05. (3) WBC cell counts, MD 0.26 (0.09, 0.43), NS; LYM cell counts, MD 0.45 (0.39, 0.51), *p* < 0.05; LYM%, MD 3.18 (2.17, 4.19), NS; CRP level, MD −8.11 (−10.41, −5.81), *p* < 0.05. (4) RR 1.42 (0.76, 2.62), *p* < 0.05.
Fu (2020b) [23]	65 (36/29) Mild, moderate A: 43.3; B: 43.7 A: 7.6; B: 8.5	(A) HM (Toujie Quwen granules, 1 packet per time for 2 times daily for 10 days, *n* = 32), plus B	(B) WM (Arbidol Hydrochloride Tablets, 200 mg + Moxifloxacin, 400 mg + Ambroxol Hydrochloride Tablets, 30 mg for 3 times daily, *n* = 33)	(1) Effective rate (2) TCM syndrome score (3) Changes in WBC cell counts, LYM cell counts, LYM% and NEU% (4) Changes in level of CRP, PCT, D-Dimer (5) Improvement of abnormalities in chest CT	(1) RR 1.35 (1.06, 1.71), *p* < 0.05. (2) Fever, MD −0.80 (−0.95, −0.65), *p* < 0.05; cough, MD −1.35 (−1.52, −1.18), *p* < 0.05; sputum production, MD −0.46 (−0.71, −0.21), *p* < 0.05; dry throat and sore throat, MD −0.48 (−0.66, −0.30), *p* < 0.05; fatigue, MD −0.34 (−0.45, −0.23), *p* < 0.05. (3) WBC cell counts, MD 0.36 (0.20, 0.52), *p* < 0.05; LYM cell counts, MD 0.26 (0.20, 0.32), *p* < 0.05; LYM%, MD 5.18 (4.11, 6.25), *p* < 0.05; NEU%, MD −4.58 (−5.81, −3.35), *p* < 0.05. (4) CRP level, MD −9.11 (−11.52, −6.70), *p* < 0.05; PCT level, MD −0.02 (−0.02, −0.01), *p* < 0.05; D-Dimer level, MD −42.50 (−84.55, −0.45), *p* < 0.05. (5) RR 1.30 (0.97, 1.74), NS.
Yu (2020) [24]	295 (171/124) Mild, moderate A: 48.2; B: 47.2 n.r.	(A) HM (Lianhua Qingwen granules, 1 packet per time for 2 times daily for 7 days, *n* = 147), plus B	(B) WM (Arbidol Hydrochloride Tablets, 200 mg + Ambroxol Hydrochloride Tablets, 30 mg for 3 times daily + Moxifloxacin tablets, 400 mg for 1 time daily, 30 mg for 3 times daily, *n* = 148)	(1) Effective rate (2) TCM syndrome score (3) Changes in WBC cell counts, LYM cell counts, CRP level and PCT level (4) Improvement of abnormalities in chest CT	(1) RR 1.25 (1.08, 1.44), *p* < 0.05. (2) Fever, MD −0.56 (−0.62, −0.50), *p* < 0.05; fatigue, MD −0.34 (−0.40, −0.28), *p* < 0.05; cough, MD −1.17 (−1.28, −1.06), *p* < 0.05; dry throat and sore throat, MD −1.34 (−1.44, −1.24), *p* < 0.05; chest tightness, MD −0.43 (−0.57, −0.29), *p* < 0.05. (3) WBC cell counts, MD 0.41 (0.33, 0.49), *p* < 0.05; LYM cell counts, MD 0.09 (0.05, 0.13), *p* < 0.05; CRP level, MD −2.00 (−3.00, −1.00), *p* < 0.05; PCT level, MD −0.01 (−0.02, −0.01), *p* < 0.05. (4) RR 1.10 (0.94, 1.30), NS.
Ye (2020) [25]	42 (7/35) Severe A: 65.0; B: 59.0 A: 9.0; B: 9.5	(A) HM (^†^ Herbal decoction, 2 times daily for 7 days, *n* = 28), plus B	(B) WM (Lopinavir/ Ritonavir, 200mg for 2 times daily, *n* = 14)	(1) Changes in the disease severity (2) Overall survival through last day of treatment (3) Proportion of patients without improvement (4) Change in serum PCT level (5) Prevalence of antibiotic use during treatment	(1) OR 0.589 (0.148, 2.352), NS. (2) OR 2.08 (0.12, 35.89), NS. (3) OR 0.44 (0.08, 2.53), NS. (4) MD 0.01 (0.00, 0.01), *p* < 0.05. (5) OR 1.84 (0.41, 8.33), NS.

CRP, C-reactive protein (mg/L); HM, herbal medicine; LYM, lymphocyte (10^9^ cells/L); n.r., not reported; PCT, procalcitonin (ng/L); TCM, traditional Chinese medicine; WBC, white blood cells (10^9^ cells/L); WM, Western medicine; NEU%: neutrophil percentage; * Diagnosis criteria was Guidelines for the Diagnosis and Treatment of 2019‒nCoVby the National Health Commission (Trial Version 5 or 6); ^†^ Compositions of the herbal decoction are provided in the Supplementary Table S1.

**Table 2:** Summary of on-going parallel randomized controlled trials (RCTs) studying the efficacy and safety of herbal medicine treatment in patients with COVID-19.

Trial Identifier	Sample Size Disease Stage No. of Trial Center	Intervention (Regimen)	Control (Regimen)	Primary Outcome Measures	Secondary Outcome Measures	Registratio*n* Date Estimate Trial Duration
NCT 04251871	150 n.r. Single	(A) HM (TCM granules, 2 times a day, for 14 days, *n* = n.r.), plus B	(B) WM (Alpha interferon (inhalation), and Lopinavir/ Ritonavir (oral) for 2 times a day, *n* = n.r.)	(1) Time to complete remission of symptoms (2) Symptoms’ change (fever and cough)	The incidence of dyspnea with low oxygen saturation level and high respiratory rate/Number of subjects who develop complications/Time to virus shedding/Time to improvement of abnormalities in chest imaging/Improvement of TCM syndrome score	5 February 2020 22 January 2020 to 22 January 2021
ChiCTR 2000029418	32 severe Single	(A) HM (n.r., *n* = 28), plus B	(B) WM (n.r., *n* = 14)	Percentage of patients progress to critically ill	Oxygenation index/Procalcitonin level/Percentage of antibiotic use	30 January 2020 3 February to 31 August 2020
ChiCTR 2000029434	240 n.r. Multiple (7)	(A) HM (Lianhua Qingwen capsules/granules, 4 capsules or 1 bag for 3 times daily, *n* = 120), plus B	(B) WM (n.r., *n* = 120)	Clinical symptoms recovery rate and recovery time (fever, fatigue, cough)	Time and rate to negativity in RT-PCR nucleic acid test/Proportion of aggravation during treatment/Rate of improvement of abnormalities in chest CT/Single symptom disappearance rate and main symptom disappearance time/Disease recovery rate/Routine blood test/Biochemical indicators	1 February 2020 1 February to 1 December 2020
ChiCTR 2000029438	100 severe or critical Single	(A) HM (n.r., *n* = 50), plus B	(B) WM (n.r., *n* = 50)	(1) CURB-65 score (2) PSI score (3) Mechanical ventilation time (4) Length of stay in hospital	Time to reduce fever /Pulmonary function/Mortality and recovery rate/Rate of multiple organ dysfunction/Time to negativity in RT-PCR nucleic acid test/Inflammation index/Incidence of AEs	1 February 2020 1 February 2020 to 1 December 2021
ChiCTR 2000029439	120 moderate Multiple (2)	(A) HM (n.r., *n* = 60), plus B	(B) WM (n.r., *n* = 60)	(1) Time to reduce fever (2) Time to negativity in RT-PCR nucleic acid test	Pulmonary function/Rate of patients’ progress to severe stage/Inflammation index/Disappearance time of gastrointestinal symptoms/TCM syndrome score/Incidence of AEs	1 February 2020 1 February to 31 December 2020
ChiCTR 2000029461	100 moderate Multiple (2)	(A) HM (n.r., *n* = 50), plus B	(B) WM (n.r., *n* = 50)	(1) Pulmonary function (2) Time to reduce fever (3) Time to negativity in RT-PCR nucleic acid test	Disappearance time of cough/Incidence of AEs/St George’s respiratory questionnaire/Modified Barthel Index/6-min walk test	2 February 2020 3 February to 31 December 2021
ChiCTR 2000029518	140 moderate or severe Single	(A) HM (n.r., *n* = 70), plus B	(B) WM (n.r., *n* = 70)	(1) Recovery time (2) Ratio and time for the moderate patients to progress to severe (3) Ratio and time for severe patients to progress to critical or death	Improvement of TCM syndrome score/Relief of clinical symptoms (fever, fatigue, gastrointestinal discomfort, etc.) and duration/Lung HRCT score improvement/Average length of hospital stay/Adverse event rate/Quality of life (SF 36)	3 February 2020 4 February to 30 April 2020
ChiCTR 2000029549	400 mild or moderate Single	(A) HM (n.r., *n* = 200), plus B	(B) WM (n.r., *n* = 200)	(1) Rate of patient’s progress to severe stage (2) Oxygenation index (3) Time to negativity in RT-PCR nucleic acid test (4) Improvement of abnormalities in chest CT	n.r.	4 February 2020 3 February to 1 May 2020
ChiCTR 2000029747	200 n.r. Multiple (4)	(A) HM (n.r., *n* = 100)	(B) WM (n.r., *n* = 100)	(1) Improvement of abnormalities in chest CT (2) Routine blood test (3) Liver and renal function (4) Improvement of TCM syndrome score	n.r.	11 February 2020 1 February 2020 to 10 February 2021
ChiCTR 2000029755	120 mild or moderate Single	(A) HM (Jinyebaidu granule, 1‒2 packets, 3 times daily, *n* = 60), plus B	(B) WM (n.r., *n* = 60)	Effective index	Safety index	12 February 2020 12 February to 30 May 2020
ChiCTR 2000029763	408 n.r. Single	(A) HM (n.r., *n* = 204), plus B	(B) WM (n.r., *n* = 204)	Rate of patients progress to severe or critical illness	Rate of isolation discharge/Improvement of TCM syndrome score/Body temperature/Blood routine test/Blood biochemical test/Improvement of abnormalities in chest imaging/Psychological outcomes	12 February 2020 12 February to 31 May 2020
ChiCTR 2000029769	40 severe Single	(A) HM (Babaodan, 6 capsules, 2 times daily, *n* = 20), plus B	(B) WM (n.r., *n* = 20)	(1) 28-day survival rate (2) Inflammatory factor levels	n.r.	13 February 2020 15 February to 30 April 2020
ChiCTR 2000029777	160 n.r. Single	(A) HM (Truncation and Torsion Formula, *n* = 80), plus B	(B) WM (n.r., *n* = 80)	(1) Responses after 14 days (recovery, improvement, turning critical, death) (2) Improvement of abnormalities in chest CT	Vital signs/Oxygenation index/Routine blood test/Inflammatory biomarkers/Major organ function/Coagulation index/APACHE II	13 February 2020 1 February to 30 June 2020
ChiCTR 2000029788	60 mild Single	(A) HM (n.r., *n* = 30), plus B	(B) WM (n.r., *n* = 30)	(1) Time to reduce fever (2) Time to negativity in RT-PCR nucleic acid test (3) Blood gas analysis (4) Improvement of TCM syndrome score	n.r.	13 February 2020 31 March to 30 December 2020
ChiCTR 2000029790	120 n.r. Single	(A) HM (n.r., *n* = 60), plus B	(B) WM (n.r., *n* = 60)	Improvement of TCM syndrome score	Rate of patients’ progress to severe or critical illness/Time to negativity in RT-PCR nucleic acid test/Time to reduce fever/Length of stay in hospital	13 February 2020 17 February to 31 October 2020
ChiCTR 2000029813	72 mild or moderate Single	(A) HM (Tanreqing, 3 capsules for 3 times daily, *n* = 36), plus B	(B) WM (n.r., *n* = 36)	(1) Time to negativity in RT-PCR nucleic acid test (2) Time to reduce fever	Arterial oxygen saturation/Rate of patients’ progress to severe or critical illness/Inflammation index (CRP)/The disappearance rate and time of cough symptoms/Clinical recovery time	14 February 2020 14 February to 14 August 2020
ChiCTR 2000029822	110 *n*.r. n.r.	(A) HM (Jinyinhua Tang, *n* = 70)	(B) Placebo (n.r., *n* = 40)	Effective rate	Time to reduce fever/Pulmonary symptoms and measure/Length of stay in hospital	14 February 2020 7 February to 30 April 2020
ChiCTR 2000029869	300 n.r. Multiple (3)	(A) HM (Baidu Jieduan formula, *n* = 150), plus B	(B) WM (n.r., *n* = 150)	(1) Responses after 14 days (recovery, improvement, turning critical, death) (2) Improvement of abnormalities in chest CT	Pneumonia symptoms/Oxygenation index/Routine blood test/Major organ function/Coagulation index/Inflammatory biomarkers	15 February 2020 1 February to 30 June 2020
ChiCTR 2000029941	200 mild, moderate, or severe Multiple (5)	(A) HM (Zhongyao Fufang granules, *n* = 100), plus B	(B) WM (n.r., *n* = 100)	Incidence of aggravation events	Total duration of disease/Length of stay in hospital/Time to total recovery/Time to negativity in RT-PCR nucleic acid test/Time from treatment to the beginning of antipyretic/Time from treatment to complete antipyretic/Improvement of abnormalities in chest imaging/Incidence of AEs	17 February 2020 1 March to 1 June 2020
ChiCTR 2000029947	200 n.r. Multiple (5)	(A) HM (Zhongyao Fufang granules, *n* = 100), plus B	(B) WM (n.r., *n* = 100)	Lung function	Total duration of disease/Time to total recovery/Incidence of AEs/Incidence of sequelae/Quality of life (SF 36)/Mental health scale	17 February 2020 1 March to 1 June 2020
ChiCTR 2000029960	100 n.r. Single	(A) HM (n.r., *n* = 70), plus B	(B) WM (n.r., *n* = 30)	Improvement of TCM syndrome score	n.r.	17 February 2020 21 February to 31 May 2020
ChiCTR 2000030034	132 n.r. Multiple (7)	(A) HM (n.r., *n* = 88), plus B	(B) WM (n.r., *n* = 44)	(1) Body temperature (2) Improvement of TCM syndrome score (3) Murray Score for Acute Lung Injury (4) Time to negativity in RT‒PCR nucleic acid test (5) MuLBSTA score	n.r.	21 February 2020 1 February to 30 June 2020
ChiCTR 2000030166	20 n.r. Single	(A) HM (Qingwen Baidu Yin granules, *n* = 10), plus B	(B) WM (Lopinavir‒ritonavir tablets + recombinant human interferon alpha 2b injection, *n* = 10)	(1) Improvement of abnormalities in chest CT (2) Nucleic acid detection of throat secretion	Body temperature/3CL Mpro of Coronavirus/Routine blood test/ Routine urine test/Liver function test/Renal function test/Routine stool test	24 February 2020 25 February to 14 May 2020
ChiCTR 2000030188	120 n.r. Single	(A) HM (n.r., *n* = 80), plus B	(B) WM (n.r., *n* = 40)	(1) Improvement of TCM syndrome score (2) Time to negativity in RT-PCR nucleic acid test (3) Cure/mortality rate	Major symptom remission time/Blood gas analysis/Indicators of inflammation (CRP, ESR)/Improvement of abnormalities in chest CT	24 February 2020 15 February to 30 March 2020
ChiCTR 2000030288	104 mild or moderate Single	(A) HM (n.r., *n* = 102), plus B	(B) WM (n.r., *n* = 102)	Time to negativity in RT-PCR nucleic acid test	The 7-point scale/Rate of patients’ progress to severe or critical illness/Routine blood test/Blood biochemical test	27 February 2020 27 February to 31 December 2020
ChiCTR 2000030469	96 moderate or severe Single	(A) HM (Liu Shen Wan, *n* = 48), plus B	(B) WM (n.r., *n* = 48)	(1) Time to reduce fever (2) Effective rate of TCM symptoms	Body temperature/Improvement of abnormalities in chest CT/Time to negativity in RT-PCR nucleic acid test/Oxygen saturation/Length of stay in hospital/Rate of patients’ progress to severe or critical illness/Improvement of TCM syndrome score/Routine blood test	2 March 2020 27 February to 27 May 2020
ChiCTR 2000030479	100 moderate Multiple (3)	(A) HM (Yiqi Huashi Jiedu Fang, *n* = 50), plus B	(B) WM (n.r., *n* = 50)	(1) Time to reduce fever (2) Time to negativity in RT-PCR nucleic acid test	Improvement of TCM syndrome score/Changes in inflammation indicators/Changes in SpO2, oxygen saturation, lymphocyte count/Time to clinical recovery/Rate of patients progress to severe or critical illness	3 March 2020 26 February 2020 to 25 February 2021
ChiCTR 2000030522	100 mild or moderateMultiple (3)	(A) HM (Ma Xing Shi Gan Tang, *n* = 50), plus B	(B) WM (n.r., *n* = 50), plus Placebo	Time to clinical recovery	Time to reduce fever/Time to negativity in RT-PCR nucleic acid test/Rate of patients’ progress to severe or critical illness/Laboratory tests (white blood cell and lymphocyte count, erythrocyte sedimentation rate, CRP/ Improvement of abnormalities in chest CT/Improvement of TCM syndrome score	5 March 2020 9 March to 9 September 2020
ChiCTR 2000030759	60 n.r. Multiple (3)	(A) HM (n.r., *n* = 56), plus B	(B) WM (n.r., *n* = 14)	(1) Time to negativity in RT-PCR nucleic acid test (2) Incidence of aggravation events (3) Time to reduce fever (4) Improvement of abnormalities in chest CT (5) Major symptom remission rate	Blood count/CRP/Blood gas analysis/Routine urine test/Blood lipid, Blood glucose, Coagulation function test/Liver function test, renal function/Myocardial enzymes/Serum procalcitonin, T-lymphocyte subsets, Interleukin	13 March 2020 15 February to 17 May 2020
ChiCTR 2000030936	2840 n.r. Multiple (71)	(A) HM (Xinguan No. 2/Xinguan No. 3, *n* = 2130), plus B	(B) WM (n.r., *n* = 710)	(1) Time to reduce fever (2) Disappearance rate of TCM symptoms	n.r.	18 March 2020 10 March to 10 May 2020
ChiCTR 2000030937	144 n.r. Multiple (6)	(A) HM (Gu Shen Ding Chuan Wan, *n* = 72), plus B	(B) WM (n.r., *n* = 72)	(1) Changes in TCM syndrome (2) Changes of fatigue assessment scale	n.r.	18 March 2020 19 March to 30 August 2020
ChiCTR 2000030988	204 mild, moderate, or severe Single	(A) HM (Hua Shi Bai Du granules, *n* = 102), plus B	(B) WM (n.r., *n* = 102)	Improvement of abnormalities in chest CT	Length of stay in hospital/Incidence of AEs	20 March 2020 20 March to 31 May 2020

AEs: adverse events; APACHE II, acute physiology and chronic health evaluation II; CRP, C-reactive protein; CURB-65, confusion, urea, respiratory rate, blood pressure, and 65 years of age or older; ESR, erythrocyte sedimentation rate; HM, herbal medicine; HRCT, high-resolution computed tomography; MuLBSTA, multi-lobular infiltration, lymphopenia, bacterial co-infection, smoking history, hypertension and age; n.r., not reported; PSI, pneumonia severity index; RT-PCR, reverse transcription polymerase chain reaction; TCM, traditional Chinese medicine; WM, Western medicine; 3CL Mpro, 3C-like proteinase.

## Supplementary Materials

The following are available online at https://www.mdpi.com/2077-0383/9/5/1583/s1, Table S1: The pattern identification and composition of herbs prescribed in Ye (2020) study.
