# Supplementary material for: Herbal Medicine for the Treatment of Coronavirus Disease 2019 (COVID-19): A Systematic Review and Meta-Analysis of Randomized Controlled Trials
Source: J Clin Med. 2020 May 23;9(5):1583. doi: 10.3390/jcm9051583 (PMC7290825; doi:10.3390/jcm9051583)
Supplement: Supplementary file 1 [file jcm-09-01583-s001.pdf]

Supplementary Table S1. The pattern identification and composition of herbs prescribed in Ye (2020) study [24].

Pattern identification (PI)	Composition of herbs
**Toxin Blocking the Lung**	Ephedrae Herba 6g, Armeniacae Semen Amarum 9g, Gypsum Fibrosum 15g, Glycyrrhizae Radix et Rhizoma 3g, Agastachis Herba 10g, Magnoliae Cortex 10g, Atractylodis Rhizoma 15g, Amomi Tsao-ko Fructus 10g, Pinelliae Rhizoma Praeparatum 9g, Poria Sclerotium 15g, Rhei Radix et Rhizoma 5g, Astragali Radix 10g, Lepidii seu Descurainiae Semen 10g, Paeoniae Radix Rubra 10g
**Blazing of both qi and nutrient**	Gypsum Fibrosum 30-60g, Anemarrhenae Rhizoma 30g, Rehmanniae Radix 30-60g, Bubali Cornu 30g, Paeoniae Radix Rubra 30g, Scrophulariae Radix 30g, Forsythiae Fructus 15g, Moutan Cortex Radicis 15g, Coptidis Rhizoma 6g, Lophatheri Herba 12g, Lepidii seu Descurainiae Semen 15g, Glycyrrhizae Radix et Rhizoma 6g

PI and herbal compositions were listed according to the Guidelines for the Diagnosis and Treatment of 2019-nCoV by the National Health Commission.
